# Supplementary material for: The cranial endocast of the Upper Devonian dipnoan ‘Chirodipterus’ australis
Source: PeerJ. 2018 Jul 6;6:e5148. doi: 10.7717/peerj.5148 (PMC6037139; doi:10.7717/peerj.5148)

**Supplementary information**

*Comments on the character matrix of Clement et al. (2016).*

The polarity of character 13 of Clement *et al.* (2016) as described in the supplementary information is incorrect in that the derived condition for the sinus superior is for it to extend dorsal to the endocranial roof as described in their text. We have corrected this in our matrix.

Supplementary Figure 1.

Single most parsimonious tree using stepwise addition with 10,000 random addition sequence replicates holding five trees at each step, with tree bisection and reconnection (TBR) enabled. Length = 36, CI = 0.639, RI = 0.667. Values at nodes are for Bremer indices.


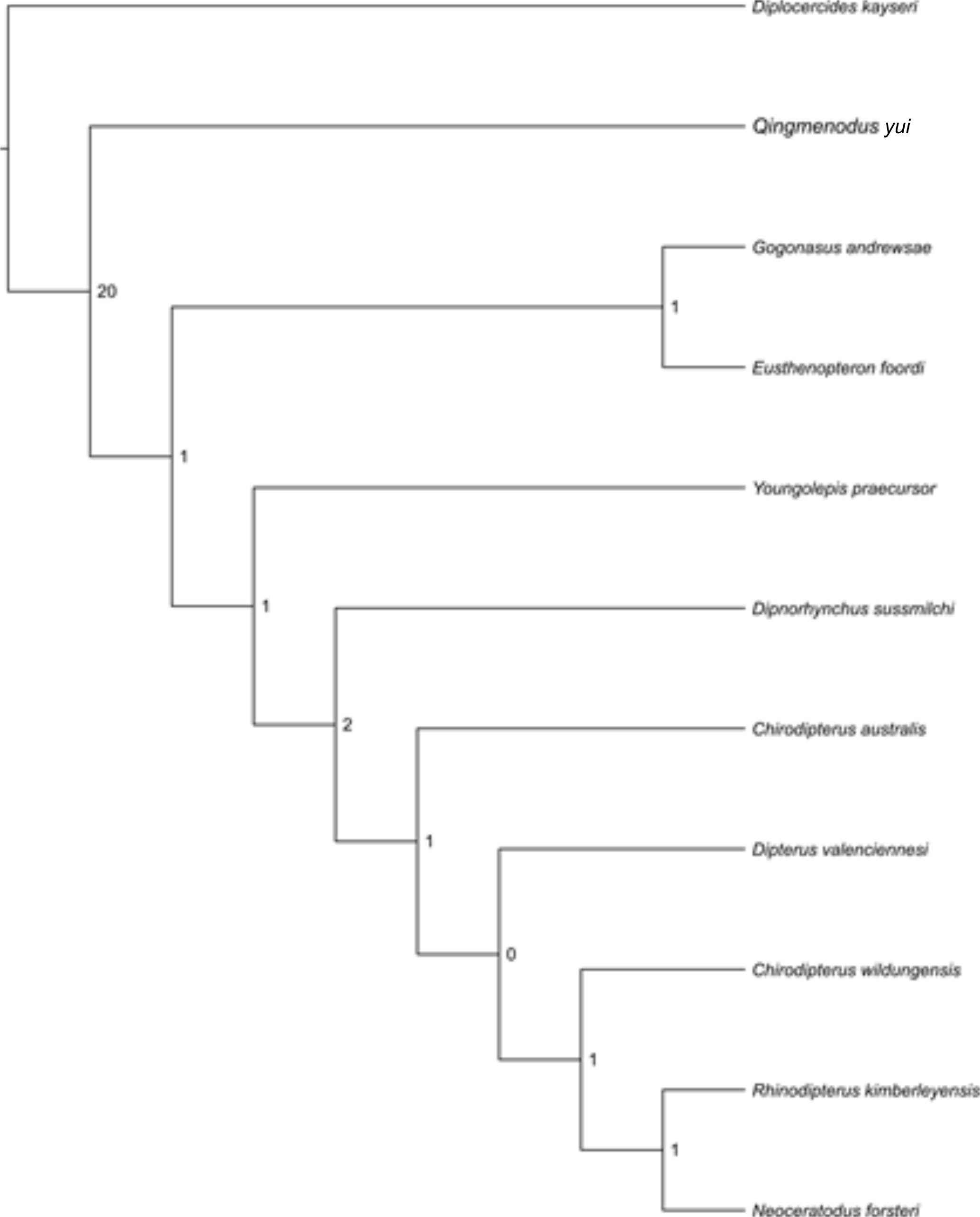


Supplementary Figure 2.

Guide to the measurements taken on the labyrinth, taken from Challands (2015).


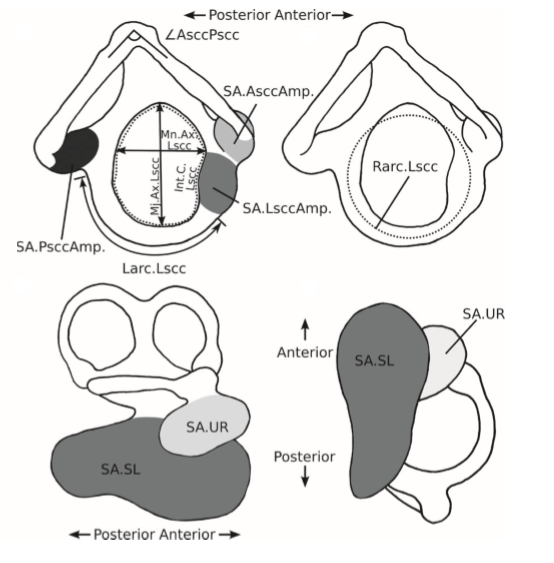

Supplement: Supplemental Information 1 — Single most parsimonious tree using stepwise addition with 10,000 random addition sequence replicates holding five trees at each step, with tree bisection and reconnection (TBR) enabled. Length = 36, CI = 0.639, RI = 0.667. Values at nodes are for Bremer indices. [file peerj-06-5148-s001.docx]
