# Supplementary material for: The cranial endocast of the Upper Devonian dipnoan ‘Chirodipterus’ australis
Source: PeerJ. 2018 Jul 6;6:e5148. doi: 10.7717/peerj.5148 (PMC6037139; doi:10.7717/peerj.5148)
Supplement: Table S1 — Measurements of the brain of Chirodipterus australis specimens NHMUK PV P56035 and NHMUK PV P56038. V.Tel.Olf, volume of the telencephalon and olfactory nerve canals; V.Di, volume of the diencephalon; V.Mes, volume of the mesencephalon; V.Rho, volume of the rhombencephalon; V.Lab, volume of the endosseous labyrinth; V.ven.exp.t, volume of the ventral expansion of the telencephalon; V.b.olf, volume of the olfactory bulbs. [file peerj-06-5148-s003.docx]

|  | Total length (mm) | Breadth (mm) | V.Tel.Olf (mm^3^) | V.Di (mm^3^) | V.Mes (mm^3^) | V.Rho (mm^3^) | V.Lab (mm^3^) | V.ven.exp.t (mm^3^) | V.b.olf (mm^3^) |
| --- | --- | --- | --- | --- | --- | --- | --- | --- | --- |
| NHMUK PV P56035 | 41.06 | 13.96 | 240 | 193 | 539 | 552 | 386 | 14.10 | 37.95 |
| NHMUK PV P56038 | 55.22 | 15.12 | 406 | 263 | 883 | 780 | 578 | 19.13 | 64.07 |

**Table 1** Measurements of the brain of *Chirodipterus australis* specimens NHMUK PV P56035 and NHMUK PV P56038. V.Tel.Olf, volume of the telencephalon and olfactory nerve canals; V.Di, volume of the diencephalon; V.Mes, volume of the mesencephalon; V.Rho, volume of the rhombencephalon; V.Lab, volume of the endosseous labyrinth; V.ven.exp.t, volume of the ventral expansion of the telencephalon; V.b.olf, volume of the olfactory bulbs.
