# Supplementary material for: The cranial endocast of the Upper Devonian dipnoan ‘Chirodipterus’ australis
Source: PeerJ. 2018 Jul 6;6:e5148. doi: 10.7717/peerj.5148 (PMC6037139; doi:10.7717/peerj.5148)
Supplement: Table S2 — Measurements of the brain of Chirodipterus australis specimens NHMUK PV P56035 and NHMUK PV P56038. “R” and “L” indicate right and left side of the endocast, respectively. ∠ nI.nI, angle between the olfactory nerve canals; ∠ nI.Tel, downward angle of olfactory nerve canals from telencephalon; na.Tel, distance from the nasal capsule to the telencephalon; na.b.olf, distance from the nasal capsule to the bottom of the olfactory bulbs; D.nI, diameter of the olfactory nerve canals; nII.wid, width of the optic nerve II; nII.dep, depth of the optic nerve II; d.end, width of the endolymphatic ducts; d.end, depth of the endolymphatic ducts. [file peerj-06-5148-s004.docx]

|  | ∠ nI.nI (º) | ∠ nI.Tel (º) | na.Tel (mm) | na.b,olf (mm) | D.nI (mm) | nII.wid (mm) | nII.dep (mm) | d.end.wid (mm) | d.end.dep (mm) |
| --- | --- | --- | --- | --- | --- | --- | --- | --- | --- |
| NHMUK PV P56035 | 73 | R: 46,  L: 30 | R: 13.18, L: 12.50 | R: 7.30,  L: 7.01 | R: 2.51,  L: 2.44 | 1.52 | 2.15 | R: 1.70,  L: 2.03 | R: 2.27,  L: 2.42 |
| NHMUK PV P56038 | 51 | R: 29,  L: 16 | R: 15.14,  L: 15.49 | R: 9.90,  L: 9.55 | R:2.85,  L: 2.52 | 1.87 | 1.99 | R: 2.83,  L: 2.72 | R: 3.98,  L: 3.34 |

**Table 2** Measurements of the brain of *Chirodipterus australis* specimens NHMUK PV P56035 and NHMUK PV P56038. "R" and "L" indicate right and left side of the endocast, respectively. ∠nI.nI, angle between the olfactory nerve canals; ∠nI.Tel, downward angle of olfactory nerve canals from telencephalon; na.Tel, distance from the nasal capsule to the telencephalon; na.b.olf, distance from the nasal capsule to the bottom of the olfactory bulbs; D.nI, diameter of the olfactory nerve canals; nII.wid, width of the optic nerve II; nII.dep, depth of the optic nerve II; d.end, width of the endolymphatic ducts; d.end, depth of the endolymphatic ducts.
