# Supplementary material for: The cranial endocast of the Upper Devonian dipnoan ‘Chirodipterus’ australis
Source: PeerJ. 2018 Jul 6;6:e5148. doi: 10.7717/peerj.5148 (PMC6037139; doi:10.7717/peerj.5148)
Supplement: Table S3 — Measurements of the semicircular canals of Chirodipterus australis specimens NHMUK PV P56035 and NHMUK PV P56038. Larc.(Assc/Pscc/Lscc), length of arc of anterior/posterior/lateral semi-circular canal; Int.C.(Assc/Pscc/Lscc), internal circumference of anterior/posterior/lateral semi-circular canal; Mj.Ax.(Assc/Pscc/Lscc), major axis of anterior/posterior/lateral semi-circular canal fenestra; Mn.Ax.(Assc/Pscc/Lscc), minor axis of anterior/posterior/lateral semi-circular canal fenestra. *Measured structure was dorso-ventrally compressed. **Dorso-ventral compression may have altered the position of the anterior ampulla and posterior ampulla. [file peerj-06-5148-s005.docx]

|  | Anterior semi-circular canal | | | | Posterior semi-circular canal | | | | Lateral semi-circular canal | | | |
| --- | --- | --- | --- | --- | --- | --- | --- | --- | --- | --- | --- | --- |
|  | Larc.Ascc (mm^2^) | Int.C.Ascc (mm^2^) | Mj.Ax.Ascc (mm^2^) | Mn.Ax.Ascc (mm^2^) | Larc.Pscc (mm^2^) | Int.C.Pscc (mm^2^) | Mj.Ax.Pscc (mm^2^) | Mn.Ax.Pscc (mm^2^) | Larc.Lscc (mm^2^) | Int.C.Lscc (mm^2^) | Mj.Ax.Lscc (mm^2^) | Mn.Ax.Lscc (mm^2^) |
| NHMUK PV P56035 right | 10.06 | 15.60 | 4.86 | 4.35 | 13.10 | 17.42 | 5.05 | 4.44 | 11.70 | 18.86 | 5.82 | 4.57 |
| NHMUK PV P56035 left | 11.03 | 16.83 | 5.05 | 4.40 | 13.60 | 18.08 | 4.71 | 4.67 | 10.06 | 18.44 | 5.67 | 4.63 |
| NHMUK PV P56038 right | 10.27* | 15.86* | 3..84* | 4.29* | 11.89 | 13.42* | 4.30* | 3.18* | 14.85 | 21.15 | 6.01 | 4.25** |

**Table 3** Measurements of the semicircular canals of *Chirodipterus australis* specimens NHMUK PV P56035 and NHMUK PV P56038. Larc.(Assc/Pscc/Lscc), length of arc of anterior/posterior/lateral semi-circular canal; Int.C.(Assc/Pscc/Lscc), internal circumference of anterior/posterior/lateral semi-circular canal; Mj.Ax.(Assc/Pscc/Lscc), major axis of anterior/posterior/lateral semi-circular canal fenestra; Mn.Ax.(Assc/Pscc/Lscc), minor axis of anterior/posterior/lateral semi-circular canal fenestra.

* Measured structure was dorso-ventrally compressed.

** Dorso-ventral compression may have altered the position of the anterior ampulla and posterior ampulla.
