# Supplementary material for: The cranial endocast of the Upper Devonian dipnoan ‘Chirodipterus’ australis
Source: PeerJ. 2018 Jul 6;6:e5148. doi: 10.7717/peerj.5148 (PMC6037139; doi:10.7717/peerj.5148)
Supplement: Table S4 — Measurements of the endosseous labyrinths of Chirodipterus australis specimens NHMUK PV P56035 and NHMUK PV P56038. The contrast in the left labyrinth was too low to confidently determine divisions between the ampullae, utriculus and sacculus. ∠ AsccPscc/Ascc.Lscc/Pscc.Lscc, angle between anterior and posterior/anterior and lateral/posterior and lateral semi-circular canals; SA.(Ascc/Pscc/Lscc).Amp., surface area of anterior/ posterior/lateral semi-circular canal ampullae; SA.UR, surface area of utricular recess; SA.SL, surface area of sacculolagenar; SA.UR/SA.SL, ratio of surface of utricular recess to surface area of sacculolagenar; V.(Ascc/Pscc/Lscc).Amp, volume of anterior/posterior/lateral semi-circular canal ampullae; V.UR, volume of the utricular recess; V.SL, volume of the sacculolagenar; V.UR/V.SL, ratio of volume of utricular recess to volume of sacculolagenar. *Measured structure was dorso-ventrally compressed. [file peerj-06-5148-s006.docx]

|  | ∠ Ascc.Pscc (º) | ∠ Ascc.Lscc (º) | ∠ Pscc.Lscc (º) | SA.Ascc.Amp. (mm^2^) | SA.Pscc.Amp. (mm^2^) | SA.Lscc.Amp. (mm^2^) | SA.UR (mm^2^) | SA.SL (mm^2^) | SA.UR/SA.SL | V.Ascc.Amp. (mm^3^) | V.Pscc.Amp. (mm^3^) | V.Lscc.Amp. (mm^3^) | V.UR (mm^3^) | V.SL (mm^3^) | V.UR/V.SL |
| --- | --- | --- | --- | --- | --- | --- | --- | --- | --- | --- | --- | --- | --- | --- | --- |
| NHMUK PV P56035 right | 108 | 121 | 106 | 50.76 | 55.27 | 56.13 | 49.53 | 370.36 | 0.13 | 11.16 | 13.46 | 10.10 | 36.96 | 143.05 | 0.26 |
| NHMUK PV P56035 left | 101 | 118 | 99 | 47.83 | 59.62 | 63.05 | 66.45 | 338.12 | 0.20 | - | - | - | - | - | - |
| NHMUK PV P56038 right | 135 | 119 | 127 | 65.28 | 39.47* | 84.21 | 130.23 | - | - | 18.04 | 14.12 | 13.10 | 88.84 | - | - |

**Table 4** Measurements of the endosseous labyrinths of *Chirodipterus australis* specimens NHMUK PV P56035 and NHMUK PV P56038. The contrast in the left labyrinth was too low to confidently determine divisions between the ampullae, utriculus and sacculus. ∠AsccPscc/Ascc.Lscc/Pscc.Lscc, angle between anterior and posterior/anterior and lateral/posterior and lateral semi-circular canals; SA.(Ascc/Pscc/Lscc).Amp., surface area of anterior/ posterior/lateral semi-circular canal ampullae; SA.UR, surface area of utricular recess; SA.SL, surface area of sacculolagenar; SA.UR/SA.SL, ratio of surface of utricular recess to surface area of sacculolagenar; V.(Ascc/Pscc/Lscc).Amp, volume of anterior/posterior/lateral semi-circular canal ampullae; V.UR, volume of the utricular recess; V.SL, volume of the sacculolagenar; V.UR/V.SL, ratio of volume of utricular recess to volume of sacculolagenar.

* Measured structure was dorso-ventrally compressed.
